# Rethinking delivery routes: oral administration of plant-derived exosome-like nanovesicles reduces the toxicity of intravenous injection

**DOI:** 10.20517/evcna.2025.164

**Published:** 2026-03-31

**Authors:** Li Chen, Bo Xiao

**Affiliations:** ^1^Department of Pharmacy, Personalized Drug Research and Therapy Key Laboratory of Sichuan Province, Sichuan Provincial People’s Hospital, School of Medicine, University of Electronic Science and Technology of China, Chengdu 610054, Sichuan, China.; ^2^Department of Biotechnology, College of Sericulture, Textile, and Biomass Sciences, Southwest University, Chongqing 400715, China.

**Keywords:** Plant-derived exosome-like nanovesicle, intravenous injection, oral administration, biosafety, immunogenicity

## Abstract

Plant-derived exosome-like nanovesicles (PELNs), as an emerging “green” nanoplatform, exhibit broad pharmacological activities, low immunogenicity, and inherent advantages as natural drug carriers. They show great potential in the pharmaceutical, cosmetic, and health supplement sectors. The clinical application of PELNs is heavily contingent on their safety profile, which is intricately linked to the administration route. This opinion compares the safety implications of the two primary routes: oral administration versus intravenous injection. Current evidence indicates that intravenous administration of PELNs triggers complement activation, immune responses, and hepatorenal toxicity; even surface engineering modifications cannot completely eliminate these risks. In contrast, oral administration of PELNs may achieve superior safety by leveraging the gastrointestinal tract’s ability to effectively reduce the immunogenic components. Based on these findings, we advocate for the prioritization of oral delivery in the future development of PELNs, given its superior safety profile for realizing their potential as natural therapeutics and drug delivery systems.

Plant-derived exosome-like nanovesicles (PELNs) are nanoscale lipid bilayer vesicles originating from various plants^[1,2]^. They are enriched with diverse biomolecules, including lipids, proteins, nucleic acids, and bioactive metabolites, which enable them to mediate intercellular communication within plants as well as cross-species signaling from plants to animals or humans^[3]^. These diverse bioactive components in PELNs underpin their broad therapeutic potential in various disease contexts, including cancer, inflammatory disease, viral infection, aging, and tissue regeneration^[4-8]^. PELNs are also regarded as a natural and safe novel drug delivery system, capable of efficiently loading and protecting small-molecule drugs or nucleic acid-based therapeutics^[9]^. Beyond the pharmaceutical field, PELNs have demonstrated considerable potential in the cosmetics and health supplement sectors^[10,11]^.

Compared with animal-derived exosomes, PELNs exhibit higher biosafety in disease therapy, as they do not carry known zoonotic pathogens^[12]^. Additionally, their wide availability, good gastrointestinal (GI) stability, and controllable production costs confer significant advantages. Despite the promise of PELNs, their translation into clinical applications presents considerable challenges, chief among them being the comprehensive assessment of their safety. This assessment is influenced by multiple factors, including plant source, extraction method, modification strategy, dosage, administration route, and microbiome, necessitating systematic evaluation in specific applications^[7,13]^. Regarding administration routes, oral delivery is a commonly used approach in PELN research, offering numerous advantages, such as ease of use, high safety, and good patient compliance^[14]^. To date, no significant systemic toxicity of oral PELNs has been reported under the tested experimental conditions. Alternatively, intravenous injection is the most widely used administration route for nanomedicines in both preclinical and clinical settings, which has also been commonly applied in PELN research. It provides rapid systemic distribution and complete bioavailability by bypassing GI and first-pass barriers, enabling precise dose control, stable blood levels, and sustained efficacy - features well-suited for acute diseases and indications requiring accurate targeting^[15-19]^. However, compared with oral delivery, intravenous injection may be associated with a higher risk of systemic adverse reactions.

In recent years, we have systematically investigated the *in vivo* safety of various PELNs and their compatibility with different administration routes. Integrating experimental results from PELNs derived from tea flower and mulberry leaf, we found that intravenous administration carried potential risks of hepatorenal toxicity and immune activation, whereas oral delivery demonstrated superior biocompatibility and safety^[20,21]^. Our previous study found that following intravenous injection of tea flower-derived exosome-like nanovesicles, mice exhibited significantly increased liver and spleen indices, accompanied by elevated serum complement 3 (C3) levels and higher alanine transaminase (ALT) and aspartate transaminase (AST) activities, indicating liver injury and immune activation^[20]^. In contrast, no significant abnormalities were observed in the oral administration group. It is hypothesized that enzymatic degradation in the GI tract may inactivate immunogenic components on the surface of PELNs, thereby reducing the systemic toxicity after absorption by the organisms. GI degradation is proposed to reduce PELN immunogenicity primarily through enzymatic processes following oral administration. Gastric acid, bile salts, and digestive enzymes preferentially degrade exogenous membrane proteins and polysaccharides that trigger immune recognition and complement activation^[22,23]^. In addition, the gut immune system is biased toward tolerance, further limiting systemic immune responses^[24]^. Together, GI degradation and the tolerogenic gut immune microenvironment may jointly mitigate immunogenicity, although this hypothesis requires further mechanistic validation.

Very recently, our study provided a more comprehensive safety assessment of intravenously administered mulberry leaf-derived exosome-like nanovesicles (MNs)^[21]^. The results revealed hepatorenal dysfunction (e.g., elevated liver indices, AST, ALT, and creatinine), immune activation (increased complement C3, spleen indices, CD3^+^/CD45^+^ lymphocytes, and white pulp hyperplasia), and mild hemolysis at high concentrations, collectively demonstrating combined hepatorenal and immune toxicity with potential hematological effects. Plant-specific proteins and polysaccharides on the surface of PELNs may directly interact with complement components, such as mannose-binding lectin, promoting C3 convertase formation and the assembly of the membrane attack complex, thereby activating the complement cascade^[25,26]^. However, the precise recognition molecules and binding patterns involved remain to be elucidated through detailed proteomic and surface characterization studies. Consistent with nanomedicine studies, intravenously administered PELNs rapidly accumulate in the liver and spleen *via* the mononuclear phagocyte system, with renal involvement in clearance^[12]^. This distribution may induce immune activation, oxidative stress, and mitochondrial dysfunction^[27,28]^, contributing to the hepatic and renal impairments, increased hepatosplenic indices, and immune responses observed in our animal studies.

To enhance the biosafety of therapeutic platforms and reduce their immunogenicity, surface engineering has emerged as a key strategy. This approach involves constructing a functional protective layer on PELNs to minimize immune recognition and activation^[5]^. To this end, Chen *et al.* further modified MNs using DSPE-PEG2000 to prepare polyethylene glycol (PEG)@MNs and systematically evaluated their biosafety^[21]^. The study demonstrated that while DSPE-PEG2000 modification improved the biosafety profile of PEG@MNs, intravenous injection still resulted in mild hepatorenal toxicity and systemic immune activation. This phenomenon may result from incomplete shielding of immunogenic epitopes by PEGylation, leaving residual plant-derived proteins or polysaccharides exposed. In addition, increasing evidence indicates that PEG can elicit anti-PEG antibodies, while *in vivo* PEG detachment or conformational changes may further compromise its immune-stealth properties^[29]^. In contrast, the oral administration group showed no significant differences from healthy controls across all measured parameters, indicating favorable biocompatibility and safety.

The comparison between oral and intravenous administration extends beyond safety to encompass delivery efficiency, practicality, and cost. Intravenous delivery enables rapid and high systemic exposure, whereas oral administration results in lower systemic levels but offers clear advantages in patient compliance, chronic use, and noninvasive application. These features align well with the potential positioning of PELNs in functional foods, nutritional interventions, and adjunctive therapies for chronic diseases. Oral formulations are more economical and scalable, as they avoid the sterile conditions and clinical settings required for injections^[30,31]^. However, oral delivery also has limitations, including low and variable bioavailability, potential GI inactivation of therapeutic components, limited targeting of distant organs, and unsuitability for certain disease indications^[32,33]^. Nevertheless, oral PELNs can exert biological effects without high systemic exposure, relying on multiple complementary pathways.

Intestinal epithelial uptake is the primary entry route for PELNs; *via* fusion, endocytosis, or receptor binding, they are internalized by M and epithelial cells, with fractions entering portal blood or lymphatics to partially bypass first-pass metabolism^[12]^. Tissue distribution studies have shown that orally administered PELNs primarily localize to digestive system tissues, with relatively weaker but detectable signals in the liver, spleen, kidney, and lung^[7,21]^. Despite limited systemic exposure, PELNs can exert significant therapeutic effects through multiple mechanisms, including local intestinal actions, rebalance of intestinal microbiota, regulation of gut-organ axes, and modulation of immune and metabolic pathways. Numerous studies have demonstrated that oral PELNs display therapeutic potential across multiple organs, including the gut, brain, bone, liver, and lungs, in various disease models (e.g., cancer, inflammatory disorder, and metabolic disease)^[34-36]^. For instance, in a breast cancer model, orally administered tea flower-derived PELNs, although slightly less effective than intravenous injection in suppressing tumor growth, still significantly inhibited orthotopic tumor growth and lung metastasis without inducing evident systemic toxicity or immune activation, whereas intravenous administration was associated with potential safety risks^[20]^. Currently, several clinical trials (e.g., NCT01668849, NCT01294072, and NCT04879810) are exploring the application of orally administered PELNs for inflammation-related diseases, metabolic disorders, and GI conditions, further confirming the clear advantages of oral administration in terms of safety, patient compliance, and feasibility for long-term applications. During oral administration, GI enzymatic digestion may attenuate surface immunogenic proteins, reducing systemic immune activation, yet the lipid bilayer and functional cargo - RNAs, proteins, and metabolites - remain largely intact^[7,37]^. This balance of preserved functionality with reduced immunogenicity underpins the concurrent safety and efficacy of oral PELNs.

The safety of PELNs is influenced not only by administration route but also by plant source and extraction method. Edible plant-derived PELNs generally show high biocompatibility and low immunogenicity, while medicinal plant PELNs, rich in active compounds, may pose higher toxicity risks, particularly at high doses or via non-oral routes^[12]^. Plant variety, growth conditions, and extraction techniques critically affect PELN composition, structure, and bioactivity; harsh or inconsistent methods can damage vesicles, alter functional cargo, and increase immunogenicity or cytotoxicity^[38]^. Standardized protocols for extraction and purification are thus essential for reproducible safety and efficacy. Currently, systematic comparative studies across plant sources and extraction methods are lacking. Large-scale production, quality control, and batch consistency remain key translational challenges^[39]^, especially for intravenous use, whereas oral administration tolerates greater variability, offering broader safety margins and lower regulatory thresholds, making it more suitable for early-stage clinical translation of PELNs.

DSPE‑PEG2000 was selected as a representative strategy due to the well‑established role of PEGylation in mitigating nanomaterial immune recognition. However, it should be noted that safety‑driven surface engineering of PELNs remains underdeveloped, and even the preferred PEG modification cannot completely eliminate the immune and organ‑related risks of intravenous administration. Alternative approaches, including membrane camouflage, ligand conjugation, and stimuli‑responsive modifications, may improve targeting or immune evasion but introduce further complexity, higher costs, potential instability, and new immunological uncertainties, all of which require rigorous systemic safety evaluation^[40]^. Therefore, increasingly complex surface modifications cannot replace a fundamental reassessment of the inherent safety limitations associated with the chosen route of administration.

Existing regulatory guidelines from regulatory agencies (such as the Food and Drug Administration, European Medicines Agency, and National Medical Products Administration) provide a framework for the safety assessment of PELNs. These guidelines highlight the need for comprehensive Investigational New Drug-phase review of PELNs, supported by long-term toxicity data. A standardized non-clinical safety evaluation framework should encompass physicochemical characterization, pharmacokinetics, toxicity profiles, and safety pharmacology studies^[16,41]^. These requirements are particularly stringent for intravenous nanocarriers, for which complement activation, cytokine release, and hemocompatibility are critical checkpoints. Consistent with these regulatory concerns, our findings of increased complement C3 levels, elevated pro-inflammatory cytokines, splenic immune cell proliferation, increased liver and spleen indices, and elevated hepatorenal parameters collectively indicate a measurable risk of systemic immune activation and hepatorenal stress upon intravenous delivery of PELNs. Therefore, PELNs that have not been thoroughly validated for safety should be used with caution for intravenous administration. Despite its risks, intravenous administration remains indispensable in scenarios requiring rapid onset, precise systemic exposure, and strict dose control, such as life-threatening acute conditions. It is also essential for applications demanding high systemic or organ-specific exposure, including certain tumor therapies and central nervous system-related studies^[19,42]^. In the context of chronic diseases, long-term interventions, and applications with higher safety requirements, oral administration offers a more favorable overall risk-benefit profile.

While existing studies generally support the good biocompatibility of PELNs, most evidence comes from short-term investigations, and long-term data remain limited^[43,44]^. Nonetheless, chronic exposure risks require assessment, as prolonged PELN administration may trigger inflammation, fibrosis, and reproductive toxicity, especially *via* accumulated lipids/proteins after intravenous delivery. Key endpoints include inflammatory biomarkers, fibrosis-related indicators, neurotoxicity, genotoxicity, toxicokinetics, and comprehensive reproductive and developmental assessments^[16,45]^. Consequently, more comprehensive and standardized toxicological studies are needed to fully characterize their safety profile and support translational development. In this context, oral administration offers inherent safety advantages: GI processing can reduce the immunogenicity of PELNs and markedly reduce systemic exposure, thereby mitigating potential risks of intravenous administration. Moreover, PELNs have been shown to maintain structural stability in GI fluids^[12]^, supporting their safety and functional integrity when delivered *via* the oral route. Taken together, these mechanistic and empirical observations suggest that orally delivered PELNs exhibit a more predictable and favorable safety profile and represent a more feasible pathway for regulatory approval and clinical translation.

## CONCLUSION

As an emerging “green” bioactive carrier and drug delivery platform, PELNs have attracted enormous attention for various biomedical applications. Their core value lies in their unique natural compositions, large-scale production, and broad pharmacological activities. The administration route profoundly influences the clinical translation of PELNs, ultimately dictating their safety and practical feasibility. Based on current research evidence, we explicitly propose that for the *in vivo* application of PELNs, oral administration is a far safer and more promising delivery strategy than intravenous injection. Although intravenous delivery can achieve systemic distribution, it carries risks of immune activation and hepatorenal toxicity that are difficult to completely avoid even with surface modifications. In contrast, oral administration leverages the natural degradative barrier of the GI tract to effectively reduce immunogenicity, while maintaining good stability and safety. Therefore, future research and applications of PELNs should prioritize oral administration, while the use of intravenous routes should be approached with caution and subjected to comprehensive preclinical safety evaluations.

## DECLARATIONS

### Copyright

© The Author(s) 2026.
